# Joining telehealth in rheumatology: a survey on the role played by personalized experience from patients’ perspective

**DOI:** 10.1186/s12913-023-09575-5

**Published:** 2023-06-22

**Authors:** Elisabetta Listorti, Lucia Ferrara, Antonella Adinolfi, Maria Chiara Gerardi, Nicola Ughi, Valeria D. Tozzi, Oscar M. Epis

**Affiliations:** 1 CERGAS SDA Bocconi, Bocconi University, Milan, Italy; 2 Rheumatology Unit, ASST Grande Ospedale Metropolitano Niguarda, Milan, Italy

**Keywords:** Telehealth, Patients, Survey, Rheumatology, Digital health, e-health

## Abstract

**Background:**

The beginning of the Covid-19 pandemic has forced many hospital departments worldwide to implement telehealth strategies for the first time. Telehealth represents the opportunity to increase value for all stakeholders, including patients and healthcare staff, but its success constitutes a challenge for all of them and particularly patients play a crucial role for their needed adherence. This study focuses on the experience of the Rheumatology Unit of Niguarda Hospital in Milan (Italy), where telehealth projects have been implemented for more than a decade with structured design and organized processes. The case study is paradigmatic because patients have experimented personalized mixes of telehealth channels, including e-mails and phone calls, Patient Reported Outcomes questionnaires, and home delivery of drugs. Given all these peculiarities, we decided to deepen patients’ perspective through three main aspects related to the adoption of telehealth: (i) the benefits perceived, (ii) the willingness to enrol in future projects, (iii) the preference on the service-mix between remote contacts and in-person visits. Most importantly, we investigated differences in the three areas among all patients based on the mix of telehealth channels experienced.

**Methods:**

We conducted a survey from November 2021 to January 2022, enrolling consecutively patients attending the Rheumatology Unit of Niguarda Hospital in Milan (Italy). Our survey comprised an introductory set of questions related to personal, social, clinical and ICT skills information, followed by the central part on telehealth. All the answers were analysed with descriptive statistics and regression models.

**Results:**

A complete response was given by 400 patients: 283 (71%) were female, 237 (59%) were 40–64 years old, 213 (53%) of them declared to work, and the disease most represented was Rheumatoid Arthritis (144 patients, 36%). Descriptive statistics and regression results revealed that (i) non-users imagined wide-ranging benefits compared to users; (ii) other things being equal, having had a more intense experience of telehealth increased the odds of accepting to participate to future projects by 3.1 times (95% C.I. 1.04–9.25), compared to non-users; (iii) the more telehealth was experienced, the higher the willingness to substitute in-person with online contacts.

**Conclusions:**

Our study contributes to enlighten the crucial role played by the telehealth experience in determining patients’ preferences.

**Supplementary Information:**

The online version contains supplementary material available at 10.1186/s12913-023-09575-5.

## Introduction

The advent of telehealth has shaped the evolution of the healthcare system during the last twenty years and has been amplified by the Covid-19 pandemic, with many hospital departments worldwide forced to implement a variety of telehealth strategies [1]. This has resulted in a plethora of scientific publications aiming at evaluating telehealth experiences [2], their effectiveness and cost-effectiveness [3], at identifying facilitators and/or barriers to their adoption [4] and the implication of telehealth from a health policy [5], a provider, and patient perspective. Studies exploring the increased use of telehealth after the Covid pandemic have all documented positive feedback about time and cost savings, acceptability of technical aspects, a high correspondence with the clinical needs of patients, reduction of missed appointment rates [6], and clinical comparability between telehealth and face-to-face care across several diseases and specialty areas [3]. Both literature and real experiences have also revealed the crucial role of patients, upon which the success of telehealth projects depends, a high level of acceptance by patients and a desire to continue in the after-pandemic future [7–9]. This high level of patient satisfaction across medical specialities calls for the need to sustain it in the long term [10], thus identifying strategical drivers of the patients’ willingness to use telehealth. Patients’ perspective needs thus to be deepened with specific research projects designed to draw insights about their beliefs and perceptions.

Given all these elements, our work aimed at exploring the patients’ perspectives on several aspects related to telehealth. In particular, we focused on the willingness of patients to enrol in telehealth projects and how this changed depending on their actual experience of telehealth. We then linked the choice of joining telehealth to two other steps that we thought might anticipate and follow the choice: the expected/perceived benefits and the preference for the combination of in-person/remote services. We focused our research on the case of the Rheumatology Unit of ASST Niguarda Hospital in Milan (Italy) for two main reasons. First, rheumatology is one of the clinical areas that have been profoundly and positively transformed by telehealth, and especially telemonitoring, since more than a decade [11, 12]. Telemonitoring requires rheumatology patients to engage in a learning experience of ICT tools and channels but also offers them promising opportunities for new follow-up strategies with fewer face-to-face visits [13], and less commitment to long-term effort of attendance in hospital wards [14, 15], even if keeping the tight control required by this chronic disease. Second, within the Rheumatology Unit of ASST Niguarda Hospital in Milan, telehealth projects have been implemented since 2010 with structured design, organised enrolment processes, and change management practices (see Appendix 1 for further details about the telehealth project). Thanks to a peculiar combination of expertise, multichannel interactions and personalised experience, it then represents a paradigmatic case study that enables us to deepen essential questions on the functioning and success of telehealth.

## Methods

### The survey

We conducted a survey to explore patients’ perspectives on several aspects related to telehealth. Our survey originated from well-known surveys [16]. Building on them, we decided to overcome the usual separation that makes surveys addressed either to telehealth users, such as in the TeleHealth Usability Questionnaire [17] or non-users, such as in the Questionnaire of Intention to use telehealth services [18], where questions to users ask about experiencing telehealth (i.e., their perceptions), while questions to non-users are more directed to their propensity to telehealth (i.e., beliefs). Our aim has been to address both groups of questions to all patients to gather insights on the impact that having experienced telehealth may have on patients’ perceptions and beliefs. Compared to a previous work with this same intention that distinguished users/non-users [19], in our research the presence of different telehealth channels allowed in-depths investigations of the impact of different degrees of involvement.

Table 1 describes the structure of our survey. It comprises an introductory set of questions related to personal, social, clinical and ICT skills information, which track the main a priori patients’ characteristics. The choice of the variables to be included has been based on previous literature [4, 20, 21] and discussion with the ward staff, which reported to us some anecdotic evidence based on their experience (mostly confirming the literature), such as the difficulty of workers with the frequency of in-person visits.

The collaboration with the staff also prepared us for the possibility of non-respondence of patients. To take advantage of this drawback, we decided to split the introductory part by inserting an initial set of questions that all patients (both respondents and non-respondents) could answer very quickly. These questions were related to personal characteristics. If patients declared not to continue the survey, a question was added about the reason for this choice. This preliminary section has enabled us to gain a picture of the whole cohort of patients attending the ward and thus to state more explicitly the possibility of under or overestimating some results depending on the characteristics of the respondents vs. the whole cohort.

The introductory part was followed by the central part on telehealth, starting with a question about the types of telehealth experienced (multiple answers enabled): any/telephone contacts/ e-mails/ Patient Reported Outcomes (PROs) filled during the visits at the ward/ PROs filled at home/ telemonitoring and home delivery of drugs. This question enabled us to characterize patients depending on the maximum degree of telehealth experienced and label them as users/non-users. For the following part, we created only a formal distinction based on the patient’s telehealth experience, i.e., questions were the same for all patients apart from the tense used, being conditional tense for non-users and past tense for users. We believe this feature enriches our survey since we obtained questions related to the propensity to telehealth and telehealth experience that allow direct comparisons between users and non-users.

The questions on telehealth explored the three areas mentioned: benefits, adherence, and preferences on service mix. By integrating comments from the ward staff and the literature on barriers and facilitators in telehealth [4, 20, 21], we also added questions on familiarity with the term telehealth, acceptability, usability, potential advantages of telehealth, and the preferred channels for the future.

The whole questionnaires were reviewed together with the ward staff, composed of physicians, nurses and health workers. Their opinions helped to fine-tune both the terminology used and the questions, to make them easily understandable by the category of patients attending the ward. The complete text of the survey is contained in the Appendix 2.

**Table 1:** Organization of the questions included in the survey

Category		Set of questions	Target
**Introductory set of questions**	**Personal**	Gender	All
		Age	
		Nationality	
		Residence	Only patients willing to complete the survey
	**Social**	Familiar status	
		Education level	
		Living alone	
		Job	
	**Clinical**	Illness (rheumatology)	
		Time from diagnosis	
		Attendance	
		Comorbidities (and attendance)	
	**ICT**	Tools	
		Online experiences	
		Competences	
**Questions on telehealth** (experimented/expected)		Familiarity with the term	
		Type of experience had	
		Acceptability, usability, potential advantages	
		Benefits	
		Channels preferred for the future	
		Future adherence	
		Service-mix	

### Data collection

We conducted the survey from November 2021 to January 2022, enrolling consecutively patients referring to the Rheumatology Unit for any reasons, thus not adopting any a-priori inclusion criteria for the enrolment to the survey. During the usual check-in of patients at the ward entrance, the staff presented the survey and the underlying project to each patient, providing the QR code to access the survey. The support also consisted in offering technological devices (i.e., tablets) to quickly open the file and assisting patients while filling the questionnaires if needed (e.g., if one question or one term was not clear to the patient). Informed consent was obtained from all subjects and all data were completely and permanently anonymized.

### Data analysis

We performed a set of descriptive statistics about the main characteristics of our cohort. We then reported the answers declared on average on the three main areas of interest (i.e., benefits, adherence, preferences on service mix). We mapped the existing differences based on the type of telehealth experience. As for the benefits, given the high number of questions on the topic, we split the respondents into users vs. non-users and we analysed the distribution of the number of benefits reported; moreover, we deepened the difference among groups for each benefit asked, performing a Chi-Square test to examine the statistical significance. As for the areas of adherence and preferences on service mix, where only one question per each area was included, graphical representations helped us to descriptively highlight differences among different types of users.

In order to draw valid conclusions that keep into account the impact of personal, social, clinical and ICT characteristics on the answers given, we ultimately ran multivariate regressions to reveal the impact of being users/non-users on the probability of reporting benefits/adherence/preferences on service-mix, considering also patients characteristics. More specifically, the information related to adherence fed into a boolean dependent variable equal to 1 if the answer to the question “Would you enrol in a telehealth project in the future?” was “Sure” or “Maybe”, 0 otherwise (“Do not think so”/”No”); as for preferences on service-mix, the boolean variable had value 1 if the person chose “Only telehealth” or “More telehealth” compared to in-person visits, 0 otherwise (“Also telehealth” or “Other”); for both these variables, logistic regressions were run. Instead, the number of benefits was calculated by summing the number of expected/experienced benefits declared, and analysed through linear regressions. Personal, social, clinical and ICT characteristics were inserted as independent variables for all the three regressions to allow cross interpretation of coefficients. Estimates were reported along with 95% confidence intervals (CI). All analyses were performed with R.

The manuscript was prepared following the CROSS reporting guideline (see Appendix 3) [22].

## Results

### Characteristics of the sample

A total of 755 patients have been enrolled for the survey (826 records, of which 71 were with no information of any type recorded). Of them, 213 stated they were not interested in questionnaires, 41 declared to have no time for the survey, and 40 said they were against telehealth.

Of the 461 patients who have decided to continue the survey, 61 gave only partial answers so they were excluded from the analysis. Hence, the cohort, composed of patients who complete the survey (from now on, full-respondents) included 400 patients who have spent a median time of 11 min (first quartile 7 min, third quartile 17 min) filling in the questionnaire.

Among the full-respondents, 71% (283) were female, 96% (384) had Italian citizenship. As for the age, the majority were between 40 and 64 years old (59%, 237 patients), 12% (48) of age 18–39, 19% (75) aged 65–74, 9% aged 75–85 (37), and 1% (3) over 85.

By having gathered this information also for those not willing to complete the survey, we can be aware of which group of patients is better/worse represented in our cohort of full-respondents. In particular, the 294 non-respondents were 75% (221) female, and 91% (268) were Italian. As for the age, 3% (9) of them were 18–39, 24% (71) in age 40–64, 33% (96) age 65–74, 37% (110) age 75–85, and 3% (8) over 85. Hence, when commenting results, attention will be paid to the over-representation of people aged 18–39 and underrepresentation of people over 75 in our cohort compared to the full cohort of patients attending the ward.

Among the full-respondents, 95% (379) declared to live in Lombardy and 53% (213) declared to work. As for the clinical characteristics, the pathologies most represented were Rheumatoid Arthritis - RA (36%, 144 patients) osteoporosis/arthrosis (21%, 82 patients), Psoriatic Arthritis - PsA (12%, 47 patients). As expected, being a chronic disease, 54% of patients (216) have received their diagnosis more than five years before. 26% of them visit the ward monthly (102), and 51% (203) have a comorbidity. Summing up the answers given to the questions about the ICT skills, only 12% (47) of patients judged them as very poor, 28% (112) as poor, 42% (169) as good and 18% (72) as excellent.

At the beginning of the set of questions about telehealth, 139 patients stated they had never heard about telehealth, even if the following answers given by this group revealed they had had several experiences.

Table 2 describes the different channels used for telehealth. By dividing patients into two main groups, we get 313 patients labelled as “non-users” and 87 patients labelled as “users”. This table explores the numerosity for each channel, in particular, (i) the number of patients that have had the experience of it, (ii) the number of patients that in the analysis are considered part of that group since that service represents the highest degree of telehealth experienced.

**Table 2:** Characteristics of the different telehealth experiences, in terms of number of patients and labels

Group of patients	Services experienced	Number of patients who have experienced that service^1^	Number of patients within the group^2^	Label	Number of patients within the group^2^
**Non-users**	None	146	146	Nothing	313
	Telephone contacts	175	138	Phone/mail	
	Direct e-mail	138			
	PROs filled within the hospital	69	29	PROs hosp	
**Users**	PROs filled at home	87	57	PROs home	87
	Tele monitoring and home delivery	30	30	Home delivery	

1. In case of services (i.e., not “none”), groups considered for this column are not mutually exclusive

2. The classification is based on the highest degree of service experienced, hence groups considered for this column are mutually exclusive

### Descriptive statistics

Results from descriptive statistics revealed existing differences between users and non-users, which can be deepened for the three main areas of interest.

#### 1. Benefits, expected or perceived

As for the benefits, the first result is the different number of benefits reported by users and non-users (Table 3). Beyond the mean number, which is 3.1 for non-users and 2.6 for users, it is useful to study the distribution of the number of benefits declared: even if in both groups 14% of patients expect no benefits, among users, 39% expect two benefits (23% among non-users), and the maximum number of benefits reported was 5, whereas it raises to 7 among non-users where the distribution is flatter.

Furthermore, it emerged that non-users imagined more benefits of various types, such as saving of travel time, immediate and direct communication allowed by telephone contact with the staff of the ward, greater emotional and psychological support received by medical staff. Some of these benefits are not related to the telehealth experience proposed by the Rheumatology ward but rather to a general idea of telehealth.

**Table 3:** Distribution of the expected or perceived benefits between users and non-users. Results represent the number of patients reporting the benefit, and in brackets the percentage of those patients within the subgroup users/non-users

Benefits	Non-users n = 313	Users n = 87	p-value
Better medical treatment experience	47 (15%)	8 (9%)	0.223
Saving of travel time	185 (59%)	37 (43%)	**0.009**
Saving of waiting time	188 (60%)	50 (57%)	0.755
Immediate and direct communication allowed by telephone contact with the staff of the ward	140 (45%)	28 (32%)	**0.048**
Convenience for receiving medicines at home (in case of biologics)	89 (28%)	24 (28%)	0.983
Greater emotional and psychological support received by medical staff	43 (14%)	3 (3%)	**0.013**

°Pearson’s Chi-squared test (bold if p < 0.05)

#### 2. Adherence to future projects

**Fig. 1:**
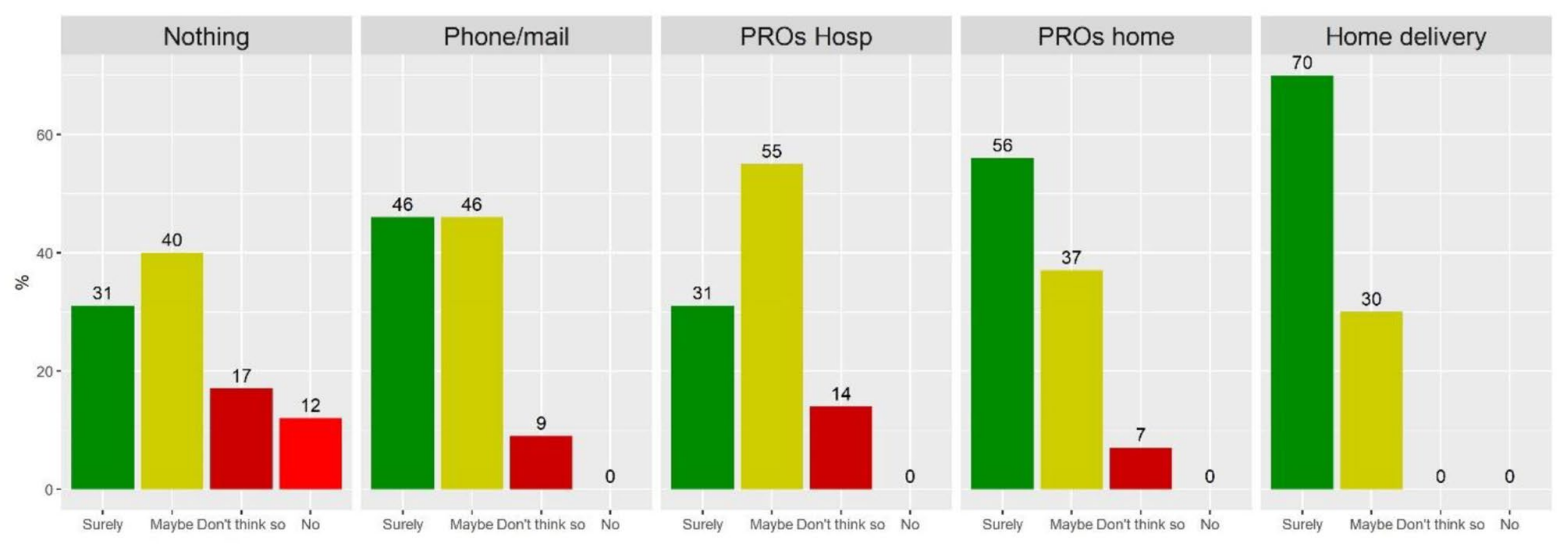
Answers to the questions on the willingness to enrol in future projects, grouped by categories of telehealth experience. Results reported as percentage of patients in each subgroup

To the question on the willingness to enrol in future project, only 4% of all respondents answered with a clear negative answer. 11% said “Don’t think so”, 42% stated “Maybe”, and 42% said “Surely”. When we split this statistic by grouping patients into users/non-users, the percentage of “Surely” increases to 61% for users, decreases to 37% among non-users. Similarly, the percentage of people saying “Don’t think so” or “No” was 5% among users, 0% among non-users. Figure 1 reports the statistic grouped by all five categories of telehealth experiences. The graph provides a qualitative but intuitive snapshot of the increasing willingness to enrol that is associated with the increasing degree of involvement into the telehealth experience.

#### 3. Expectations on the personalization

**Fig. 2:**
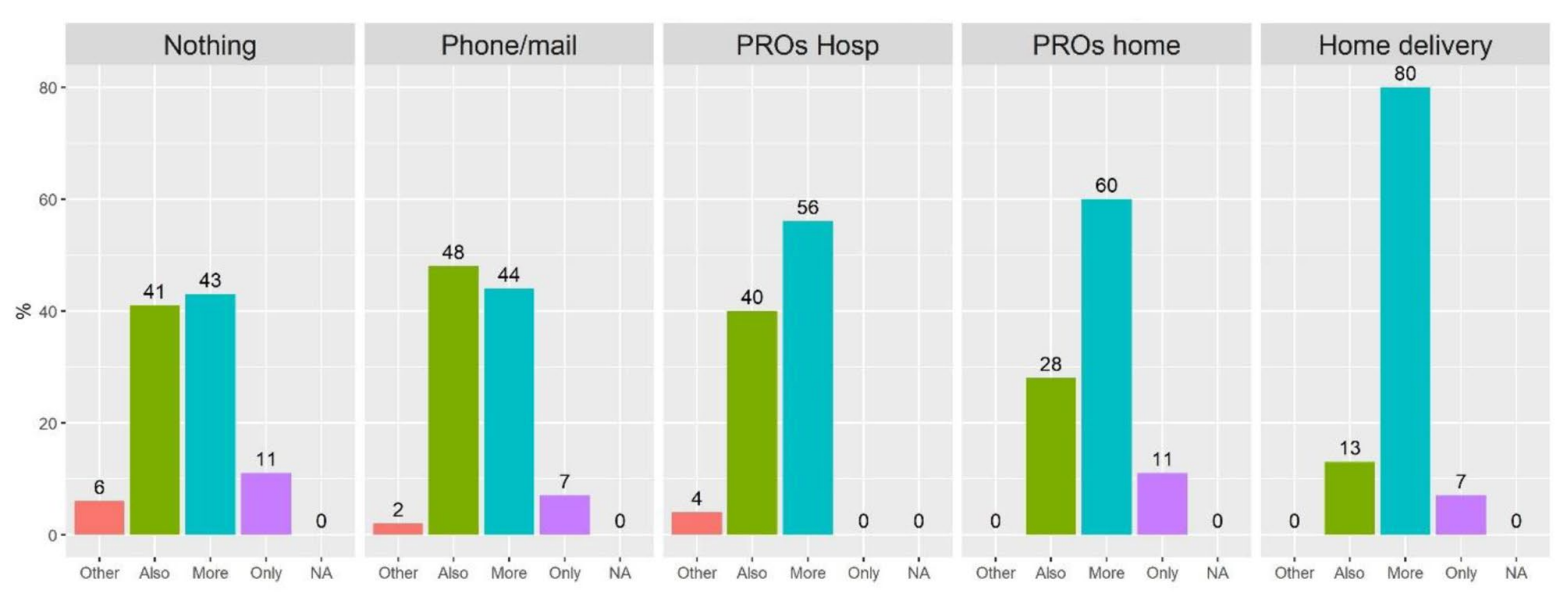
Answers to the questions on the type of personalization desired, grouped by categories of telehealth experience. Results reported as percentage of patients in each subgroup

A similar mechanism of “the more experienced-the more willingness” appears also for the type of personalization desired by patients (Fig. 2, question “How much on-line contacts would you prefer compared to in-person visits?”): 80% of patients with experience of home delivery declare to prefer more telehealth, compared to 43% among never users. Moreover, the general answer “Other” to the same question goes disappearing as people get involved in telehealth.

### Multivariate logistic regression

Multivariate logistic regression models further confirmed the descriptive results, as reported in Table 4. As for the willingness to participate to future telehealth projects, all things considered, having had a more intense experience of telehealth increases the odds of accepting by 3.1 times (95% C.I. 1.04–9.25), compared to non-users. Furthermore, the more telehealth is experienced, the higher the willingness to substitute in-person visits with online contacts with an odds of 2.722 (95% CI 1.514–5.067). On the other side, having experienced more telehealth channels drives to a lower number of benefits reported estimated as -0.722 (95% CI= -1.133- -0.311).

The multivariate regression allows also to explore the impact of other influencing factors documented by the literature. More specifically, our results report a significant impact of being a worker as for the willingness to enrol in future projects, as well as of having been diagnosed for more than five years. Having good ICT skills plays a role in increasing the benefits reported and the odds of accepting new telehealth projects.

**Table 4:** Results from multivariate regressions, expressed as beta coefficients for linear regression

Independent variables	Dependent variables		
	Number of benefits *Regression (1)*	Willingness to enrol in future projects *Regression (2)*	Type of personalization: all or most on-line contacts *Regression (3)*
**Gender: male**	0.15 (-0.218, 0.518)	1.169 (0.580, 2.448)	1.274 (0.755, 2.164)
**Age < 40**	0.261 (-0.193, 0.714)	1.419 (0.696, 2.920)	0.750 (0.379, 1.469)
**Foreign citizenship**	-0.276 (-1.112, 0.560)	2.407 (0.427, 45.460)	0.853 (0.273, 2.616)
**Low education**	0.267 (-0.536, 1.070)	0.825 (0.274, 2.688)	1.161 (0.327, 4.102)
**Residence in Lombardy**	0.196 (-0.526, 0.919)	1.600 (0.392, 11.008)	2.226 (0.789, 7.291)
**Worker**	0.155 (-0.265, 0.575)	2.990** **(1.361, 6.671)**	1.517 (0.836, 2.764)
**Living alone**	-0.153 (-0.574, 0.267)	0.799 (0.387, 1.728)	0.623 (0.339, 1.140)
**Good ICT skills**	0.609** **(0.234, 0.984)**	1.974* **(1.010, 3.896)**	1.447 (0.853, 2.458)
**Diagnosis from more than 5 years**	0.170 (-0.173, 0.513)	2.051* **(1.102, 3.882)**	1.441 (0.886, 2.355)
**High frequency of visits in hospital**	0.070 (-0.279, 0.420)	1.115 (0.592, 2.160)	0.947 (0.575, 1.563)
**User (vs. no user)**	-0.722*** **(-1.133, -0.311)**	3.064* **(1.137, 10.719)**	2.722** **(1.514, 5.067)**

(1) Odds Ratios for logistic regressions (2) and (3), and 95% Confidence Intervals (* p < 0.05, **p < 0.01, ***p < 0.001)

## Discussion

The widespread phenomenon of telehealth calls for further research aimed at clarifying its functioning and strategies for success. Policy makers worldwide are forced to implement telehealth projects that need to be built on the lessons learned from previous experiences. Nevertheless, what emerges from the recent literature on telehealth is a twofold finding: the uniqueness of each telehealth experience, which is necessarily influenced by the specific context where it originates; and the difficulty in generalising the strategic drivers that guarantee experiments of telehealth to be successful. These two points require the development of real evaluations of previous experiences, which accounts for all contextual factors affecting the output and thus enable to draw insights to be disseminated. However, the methodology of real evaluations is based on the clear identification of the contextual factors of the experiences, which is easier when these experiences are characterized by a high level of preparedness, a well-structured organization and a story of a number of attempts that have conducted to long-term working solutions.

With this perspective, our case study at the Rheumatology Unit of ASST Niguarda Hospital, thanks to its grounded experience, represents an opportunity to highlight successful elements that may be reproduced elsewhere. Referring to the main theoretical models of the literature, such as the Technology Acceptance Model (TAM) and the Unified theory of acceptance and use of technology (UTAUT), we analysed the case study by focusing on the concept of behavioural intention. We specifically aim to uncover the bidirectional linkage with the Actual System Use [23].

Our analysis shows four main results and implications. First, the principal result that emerges is the crucial role played by patients’ experience in determining their approach to telehealth, i.e., the more telehealth has been experimented in the past, the more the propensity to join telehealth projects in the future. In particular, departing from several previous works that have considered either telehealth users or never users, we deepened the impact that the type of telehealth experience (whether intense or absent, considering its degree of pervasiveness) has on the patients’ approach to telehealth as a whole. Indeed our data shows that users, compared to non-users, tend to (i) expect fewer benefits, but more realistic; (ii) have a higher propensity to enrol in future telehealth projects; (iii) be more likely to substitute in-person visits with on-line visits. This result holds once considered all the factors documented by the literature as affecting patients’ propensity towards telehealth.

Second, our analysis contributes to the debate on the predictive factors toward joining telehealth. In particular, personal ICT skills, being a worker and having been diagnosed for a long time were revealed to significantly affect patients’ preferences. At the same time, population age was not a predictive factor. The role of age on the intention to join or not telehealth projects is highly debated in the literature and is still controversial. If on one hand it is well discussed in the literature the digital divide of older people, people with low levels of income, education and employment [24], it is now emerging that older people’s use of digital tools is less influenced by socioeconomic disadvantage and more linked to practical issues of capacity, comfort and ease of use. Our result shows that age was not a predictive factor affecting patients’ propensity toward telehealth, and this is in line with previous results documented by a national survey [25], and by a recent US study which demonstrated that older people who had experience with telehealth enjoyed the experience and were more willing to use telehealth in the future [26]. It could be then that also for this group of patients, their previous experience with telehealth plays a crucial role in their willingness to adopt telehealth in the future. This mechanism may also justify the high number of older people that did not accept to participate in our survey, assuming that they have refused to participate because they had never had any telehealth experience before. In this sense, our study supports the thesis that for older people the barrier to joining telehealth may be more related to digital literacy and experience than deprivation or disadvantage.

Third, our study contributes to the literature by considering a group of patients who have had a variety of telehealth experiences as a result of personalisation. The Rheumatology Unit offers the single patient the opportunity to customize the kind of interactions with the healthcare system: the final decision on the mix of channels used relies on the patient and can be tailored (i.e., based on his preparedness, on the availability of the device and the specific situation). With this approach, patients can gradually experiment with personalised mixes of telehealth channels, including e-mails and telephone contacts with doctors, patient reported outcomes questionnaires filled within the ward or at home and home delivery of drugs.

This finding deeply recalls the theme of personalization, patients’ empowerment, engagement and involvement. First, the personalisation of telehealth may combine patients’ clinical needs with patients’ preferences and, ultimately, professionals’ availabilities. Second, patients need to understand, accept and experiment a treatment to gain awareness of what is happening to them. Also, patient education and digital health literacy requires to be considered when implementing telehealth projects. These mechanisms and activities increase their empowerment and engagement, adherence and ultimately the efficacy of the care pathway. The combination between digital health, integrated care, and patients’ empowerment may represent a key strategy for the challenge faced by the healthcare systems worldwide [27]. Eventually, patients also need to be involved in the choices regarding their health and in developing such telehealth solutions. As discussed in the literature [28, 29], the development of telehealth solutions requires a proper engagement of all stakeholders and end-users in a process of co-creation and co-design. The experience of the Rheumatology Unit of ASS Niguarda Hospital is a good example of this end, as the App was co-designed with patients and professionals.

Finally, our study highlights that telehealth represents the opportunity to increase value for all stakeholders, but its success constitutes a challenge for all of them. Telehealth requires ensuring high quality and continuity of remote monitoring, which have implications on the management and work-life of the whole staff of the rheumatology units. In fact, even though telehealth can also represent the opportunity for better case management for physicians, nurses and administrative staff, it also calls for a reorganisation of workflows and processes [30, 31].

Our survey also reveals a set of open questions for future research, of which three appear of particular interest. As reported in the results, a group of patients stated they had never heard about telehealth, even if their following answers revealed they had had several experiences. This inconsistency suggests a problem with terminology, raising doubts about the spread of a recognized culture of telehealth. On the other side, ten users declared to be willing to enrol in future telehealth projects, but did not tick any benefit among the ones included in the survey, suggesting that there are positive sides of telehealth that still need to be identified. Third, the Rheumatology Unit piloted the telehealth project in 2010 as part of a Regional project even without a formal recognition and reimbursement of telehealth. In Italy, between 2020 and 2021, more than half of the regions have defined legislative criteria to regulate and reimburse the new telehealth initiatives, thus highlighting the willingness and necessity to engage in this field [32]. However, the development of telehealth forces the entire system to reflect on three interconnected dimensions: (i) the clinical dimension, i.e., which patients are more likely to enrol in telehealth and can maximize their outcome by mixing face-to-face and online interactions; (ii) the economic dimension, i.e., what are the economic implications of telehealth from a provider and societal perspective; and (iii) the managerial dimension, i.e., how telehealth will affect the reorganization of healthcare process and the care pathway.

Our study presents strengths and limitations. Beyond the mentioned peculiar characteristics of the case study, the use of a survey enables to collect information about patients’ perspective in a direct way. The data provided by patients related to personal, clinical and social characteristics have been exploited within the analysis to confirm literature findings on factors affecting the propensity to telehealth and to obtain more robust results that can be extended to other cohorts. Furthermore, an important enriching element has been the interaction with the ward staff through workshops and interviews, where they have shared their opinions on the telehealth experiences developed so far. The continuous combination of literature, research findings and anecdotal evidence has allowed us to achieve increasing knowledge about the phenomenon. Nevertheless, this case study could present specific features that make the results only partially valid at an external level. Moreover, further research is needed to deepen organizational and economic considerations that enable the reproducibility of similar projects.

## Conclusion

Our study contributes to enlighten the crucial role played by the telehealth experience in determining patients’ preferences. Results from a survey with 400 respondents conducted in the rheumatology ward of the Niguarda Hospital (Milan, Italy) have revealed that, based on the type of telehealth experience that patients had, they declare a different propensity to enrol in future telehealth projects. On one side, being a telehealth user makes patients more aware of the realistic benefits to be expected from the specific telehealth experience. On the other side, it appears that the more telehealth is experienced, the higher the willingness to adhere to future projects and to increase remote contacts. The importance of these results is supported by the combination of expertise, multichannel interactions and personalization experience that characterizes the case study analysed.

## Electronic supplementary material

Below is the link to the electronic supplementary material.

Supplementary Material 1

## Data Availability

The data that support the findings of this study are of property of the funding body and were used under agreement for the current study. For this reason, they are not publicly available but are available from the corresponding author on reasonable request.

## References

[1] WHO. Implementing telemedicine services during COVID-19: guiding principles and considerations for a stepwise approach. WHO Regional Office for the Western Pacific; 2020.

[2] Gonçalves-Bradley DC, Maria ARJ, Ricci-Cabello I, Villanueva G, Fønhus MS, Glenton C et al. Mobile technologies to support healthcare provider to healthcare provider communication and management of care. Cochrane Database Syst Rev. 2020. 10.1002/14651858.CD012927.pub2 PMC7437392 32813281

[3] Eze ND, Mateus C, Cravo Oliveira Hashiguchi T (2020). Telemedicine in the OECD: an umbrella review of clinical and cost-effectiveness, patient experience and implementation. PLoS ONE.

[4] Almathami HKY, Win KT, Vlahu-Gjorgievska E (2020). Barriers and facilitators that influence telemedicine-based, real-time, online consultation at patients’ homes: systematic literature review. J Med Internet Res.

[5] Kruse CS, Williams K, Bohls J, Shamsi W (2021). Telemedicine and health policy: a systematic review. Health Policy Technol.

[6] Pogorzelska K, Chlabicz S (2022). Patient satisfaction with telemedicine during the COVID-19 Pandemic-A systematic review. Int J Environ Res Public Health.

[7] Aashima NM, Sharma R (2021). A review of patient satisfaction and experience with telemedicine: a virtual solution during and beyond COVID-19 pandemic. Telemed E-Health.

[8] De Simone S, Franco M, Servillo G, Vargas M (2022). Implementations and strategies of telehealth during COVID-19 outbreak: a systematic review. BMC Health Serv Res.

[9] Giansanti D, Morone G, Loreti A, Germanotta M, Aprile I. In: Healthcare, editor. A narrative review of the Launch and the Deployment of Telemedicine in Italy during the COVID-19 pandemic. MDPI; 2022. p. 415. 10.3390/healthcare10030415 PMC8955340 35326894

[10] Nittari G, Savva D, Tomassoni D, Tayebati SK, Amenta F (2022). Telemedicine in the COVID-19 era: a narrative review based on current evidence. Int J Environ Res Public Health.

[11] Jackson LE, Edgil TA, Hill B, Owensby JK, Smith CH, Singh JA et al. Telemedicine in rheumatology care: a systematic review. Seminars in arthritis and Rheumatism. Elsevier; 2022. 152045. 10.1016/j.semarthrit.2022.152045 35843158

[12] McDougall JA, Ferucci ED, Glover J, Fraenkel L (2017). Telerheumatology: a systematic review. Arthritis Care Res.

[13] Silvagni E, Sakellariou G, Bortoluzzi A, Giollo A, Ughi N, Vultaggio L (2021). One year in review 2021: novelties in the treatment of rheumatoid arthritis. Clin Exp Rheumatol.

[14] Bakker MF, Jacobs JWG, Verstappen SMM, Bijlsma JWJ (2007). Tight control in the treatment of rheumatoid arthritis: efficacy and feasibility. Ann Rheum Dis.

[15] Schipper LG, Van Hulst LT, Grol R, Van Riel PL, Hulscher ME, Fransen J (2010). Meta-analysis of tight control strategies in rheumatoid arthritis: protocolized treatment has additional value with respect to the clinical outcome. Rheumatology.

[16] Hajesmaeel-Gohari S, Bahaadinbeigy K (2021). The most used questionnaires for evaluating telemedicine services. BMC Med Inform Decis Mak.

[17] Parmanto B, Lewis AN, Graham KM, Bertolet MH (2016). Development of the telehealth usability questionnaire (TUQ). Int J Telerehabilitation.

[18] Ghaddar S, Vatcheva KP, Alvarado SG, Mykyta L (2020). Understanding the intention to use telehealth services in underserved hispanic border communities: cross-sectional study. J Med Internet Res.

[19] Ferucci ED, Holck P, Day GM, Choromanski TL, Freeman SL (2020). Factors associated with use of telemedicine for follow-up of rheumatoid arthritis. Arthritis Care Res.

[20] Kong SS, Otalora Rojas LA, Ashour A, Robinson M, Hosterman T, Bhanusali N (2021). Ability and willingness to utilize telemedicine among rheumatology patients—a cross-sectional survey. Clin Rheumatol.

[21] Ross J, Stevenson F, Lau R, Murray E (2016). Factors that influence the implementation of e-health: a systematic review of systematic reviews (an update). Implement Sci.

[22] Sharma A, Minh Duc NT, Luu Lam Thang T, Nam NH, Ng SJ, Abbas KS (2021). A consensus-based checklist for reporting of survey studies (CROSS). J Gen Intern Med.

[23] Venkatesh V, Thong JY, Xu X (2016). Unified theory of acceptance and use of technology: a synthesis and the road ahead. J Assoc Inf Syst.

[24] Dykgraaf SH, Desborough JL, Sturgiss EA, Parkinson A, Dut GM, Kidd M. Older people, the digital divide and use of telehealth during the COVID-19 pandemic. Aust J Gen Pract. 2022;51. 10.31128/AJGP-03-22-6358 36045630

[25] Cavagna L, Zanframundo G, Codullo V, Pisu MG, Caporali R, Montecucco C (2021). Telemedicine in rheumatology: a reliable approach beyond the pandemic. Rheumatology.

[26] Bhatia R, Gilliam E, Aliberti G, Pinheiro A, Karamourtopoulos M, Davis RB (2022). Older adults’ perspectives on primary care telemedicine during the COVID-19 pandemic. J Am Geriatr Soc.

[27] Knight A, Burdett T (2021). Achieving integrated care: the need for digital empowerment. Perspect Public Health.

[28] Tarricone R, Cucciniello M, Armeni P, Petracca F, Desouza KC, Hall LK et al. Mobile Health Divide Between Clinicians and Patients in Cancer Care: Results From a Cross-Sectional International Survey. JMIR MHealth UHealth. 2019;7. 10.2196/13584 PMC6754682 31493318

[29] Tarricone R, Petracca F, Cucciniello M, Ciani O (2022). Recommendations for developing a lifecycle, multidimensional assessment framework for mobile medical apps. Health Econ.

[30] Muehlensiepen F, Knitza J, Marquardt W, May S, Krusche M, Hueber A (2021). Opportunities and barriers of telemedicine in rheumatology: a participatory, mixed-methods study. Int J Environ Res Public Health.

[31] Nguyen M, Waller M, Pandya A, Portnoy J (2020). A review of patient and provider satisfaction with telemedicine. Curr Allergy Asthma Rep.

[32] Bobini M, Boscolo PR, Tozzi VD, Tarricone R (2021). La telemedicina e i processi di gestione del cambiamento nelle aziende sanitarie. Rapporto OASI.

