# Supplementary material for: Joining telehealth in rheumatology: a survey on the role played by personalized experience from patients’ perspective
Source: BMC Health Serv Res. 2023 Jun 22;23:682. doi: 10.1186/s12913-023-09575-5 (PMC10288781; doi:10.1186/s12913-023-09575-5)
Supplement: Supplementary file 1 — Supplementary Material 1 [file 12913_2023_9575_MOESM1_ESM.docx]

**Appendix 1. Telehealth projects at the Rheumatology Unit of Niguarda Hospital**

Since 2010, the rheumatology unit has engaged patients for reporting their status of health using Patient Reported Outcomes (PROs). The first telehealth project dates back to 2011, when an App (iArPlus) was introduced for the collection and management of clinical data during the examinations of patients with Rheumatoid Arthritis (RA), Psoriatic Arthritis (PsA) and Ankylosing Spondylitis (AS) and for the remote monitoring of patient conditions. Patients in fact had the possibility to enter data on PROs remotely or before the visit, with the support of the Rheumatology Unit staff. An *ad-hoc* evaluation found high level of agreement between electronic and paper-and-pencil PROs responses [1]. From the beginning, the iArPlus platform has been accessible both through a link on the Web and through a dedicated app for the tablet.

In 2019 the project consolidated and added the home-delivery of biological drugs for patients with stable disease. Patients had the possibility to enter data on PROs remotely (every fortnight) and, based on the evaluation of PROs, if there were no critical issues, the clinicians were able to postpone the outpatient visit, and the drug was delivered directly to the patients’ home. During the first wave of the Covid-19 pandemic (March-May 2020), given the lockdown and increasingly distressed healthcare situation in northern Italy, the project was further enhanced and all patients with RA, PsA, and AS being treated at the Unit with specific biological drugs were followed remotely [2].

As a result, since 2011 patients have had the opportunity to gradually experiment with personalised mixes of telehealth channels, including e-mails and telephone contacts with doctors, PROs questionnaires filled within the ward or at home and home delivery of drugs. While the possibility to use the app iARplus and home delivery of drugs depended on the illness, and ultimately on patients’ will, the use of e-mails and telephone contacts was available for all patients at all clinical stages. This combination of expertise, multichannel interactions and personalised experience differentiates from other previous experiences in the field, that mostly refer to the post-pandemic period [3, 4]. For these reasons, the experience raises interest both in the organizational aspects and the clinical consequences. As for the organization, the telehealth project reduced the number of clinical visits by 69.9% while maintaining therapeutic continuity. As for patient’s health, a study published last year reported no clinically significant differences between PROs before and after three months of using either telehealth or in-person visits [2]. The two results document the success of this experience.

**References**

1. Epis, O.M., Casu, C., Belloli, L., Schito, E., Filippini, D., Muscarà, M., Gentile, M.G., Cagnone, P.C.P., Venerelli, C., Sonnati, M. and Schiavetti, I., 2016. Pixel or paper? Validation of a mobile technology for collecting patient-reported outcomes in rheumatoid arthritis. JMIR Research Protocols, 5(4), p.e5631.
2. Chevallard, M., Belloli, L., Ughi, N., Adinolfi, A., Casu, C., Di Cicco, M., Filippini, D.A., Muscarà, M., Schito, E., Verduci, E. and Gentile, M.G., 2021. Use of telemedicine during the COVID-19 pandemic in patients with inflammatory arthritis: a retrospective study on feasibility and impact on patient-reported outcomes in a real-life setting. Rheumatology international, 41(7), pp.1253-1261.
3. Opinc, A., Łukasik, Z. and Makowska, J., 2020. The attitude of Polish rheumatology patients towards telemedicine in the age of the COVID-19 pandemic. Reumatologia, 58(3), p.134.
4. Shenoy, P., Ahmed, S., Paul, A., Skaria, T.G., Joby, J. and Alias, B., 2020. Switching to teleconsultation for rheumatology in the wake of the COVID-19 pandemic: feasibility and patient response in India. Clinical rheumatology, 39(9), pp.2757-2762.

**Appendix 2. Questionnaire "Towards multichannel models: an innovative model for taking care of patients with rheumatoid arthritis"**

The rheumatology department of the Niguarda hospital is involved in a collaboration with anonymous and the Galapagos company. The goal of the project is to investigate the topic of telemedicine. Telemedicine is the set of medical and IT techniques that allow the treatment of a patient at a distance or, more generally, to provide health services at a distance. We would like to know your opinion and experience with it, and for this we ask you to dedicate 15 minutes of your time to us. Whether you have never heard of it or you are an expert on the subject, telling us what you think will help improve the quality and innovation of the health system.

1. Sex
   - Male
   - Female
2. Which category best represents your age?
   - Under 18
   - 18-39
   - 40-64
   - 65-74
   - 75-85
   - Over 85
3. Nationality
   - Italian
   - Other (specificy)
4. We are administering a questionnaire about your experience and propensity for telemedicine. Telemedicine is the set of medical and IT techniques that allow the treatment of a patient at a distance or more generally to provide health services at a distance. Do you wish to continue?
   - Yes
   - No, because I don't have time
   - No, because I am against telemedicine
   - No, I am not interested in answering questionnaires

**For those who answered YES to question 4**

1. What is your family status?
   - Married
   - Living together
   - Widowed
   - Divorced
   - Separated
   - Single
2. Who lives in the house with you? (multiple answers are possible):
   - Nobody, I live alone
   - Spouse/ Partner
   - Children/ Children up to 14 years
   - Children, other relatives or friends aged 15-64
   - Other relatives or friends aged 65 and over
3. What is the highest level of education you have ever obtained?
   - Primary School
   - Intermediary School
   - Secondary School
   - Degree
   - Master
   - PhD
   - Other
4. Which of the following categories best characterizes your employment status?
   - Employee, work 1-39 hours/ week
   - Employee, I work 40 hours or more/ week
   - Unemployed, looking for a job
   - Unemployed, NOT looking for a job
   - Retired
   - Unable to work
5. What is your municipality of residence? ________
6. For which of these diseases are you referring to this clinic?
   - Rheumatoid Arthritis
   - Psoriatic Arthritis
   - Spondyloarthritis/ Ankylosing Spondylitis
   - Scleroderma/ Systemic sclerosis
   - Systemic lupus erythematosus
   - Sjogren's syndrome
   - Osteoporosis/ arthrosis
   - Other ________
7. When did you receive the diagnosis?
   - Less than a year ago
   - 1-5 years ago
   - More than 5 years ago
   - I don't remember
8. How often do you go to the rheumatology ward for visits?
   - Once or twice a year
   - Three to four times a year
   - Monthly or almost every month
   - More than once a month
9. Select one or more of these diseases if you have been diagnosed or confirmed by a doctor:
   - Diabetes
   - Renal failure
   - High blood pressure
   - Chronic bronchitis, emphysema, respiratory failure, bronchial asthma
   - Myocardial infarction or other heart disease
   - Cancers (including leukemias and lymphomas)
   - Chronic liver disease, cirrhosis
   - Other_______
10. **(in case of at least one flag to question 13)** How often do you go to hospital or to a specialist clinic visits for non-rheumatological diseases?
    - Once or twice a year
    - Three to four times a year
    - Monthly or almost every month
    - More than once a month

We will now ask you questions related to your familiarity with technology tools.

1. Which of the following tools do you have to make calls or video calls? (indicate all the tools available)
   - Desk phone
   - Mobile phone
   - Mobile phone with the possibility of making video calls
   - Tablet
   - Computer
   - None
   - Other
2. Have you ever gone online to browse the Internet or send /receive e-mails?
   - Yes
   - No
3. How would you rate your computer skills when using the Internet:
   - Excellent
   - Good
   - Poor
   - Bad

The next questions will focus on the topic of **telemedicine**. Telemedicine is the set of medical and IT techniques that allow the treatment of a patient at a distance or more generally to provide health services at a distance. Examples are televisits (i.e., the professional interacts remotely with the patient in real time via call or video call); remote reporting (i.e., sharing of medical reports remotely); telemonitoring (i.e., remote monitoring of clinical parameters through questionnaires or wearable medical devices); telerehabilitation (i.e., the rehabilitation activity carried out remotely).

1. Have you ever heard of telemedicine?
   - Yes
   - No
2. Since you have been treated in the rheumatology department, which services have you used (it is also possible to select more than one answer):
   - I contacted the medical staff by phone
   - I contacted the medical staff by e-mail
   - I filled out the questionnaires in hospital via the app with the support of the ward staff
   - I filled out the questionnaires at home via the app
   - I filled out the questionnaires at home via the app and received the drug at home
   - I filled out the questionnaires via the app to receive the drug at home, but I did not receive it
   - Other __________

**Questionnaire reserved for users (identified by question 19)**

1. In which period did you fill out the questionnaires at home (and possibly received the drug at home):
   - Before and after February 2020
   - Only before February 2020
   - Only after February 2020
   - I don't remember

**To answer the next questions, focus on your experience with the rheumatology department which involved completing questionnaires at home (hereinafter, "telemedicine"), and think about the services you have used.**

1. On a scale of 1 (disagree) to 5 (completely agree), indicate what you think of the following statements:
   - It was easy to use the system to fill the questionnaires at home
   - It was easy to learn how to use the system to fill the questionnaires at home
   - Telemedicine improves access to health services
   - Telemedicine has allowed me to receive specialized care that I otherwise would not have been able to access
   - Telemedicine has saved me the travel time to go to the hospital
   - Telemedicine meets my health needs
   - I believe that communications with staff through the telemedicine system are equivalent to communications in face-to-face meetings
   - I enjoy communicating with medical staff using telemedicine tools
   - I believe that telemedicine tools improve my ability to talk to doctors (eg. Asking questions, telling concerns, etc.)
   - Telemedicine is an acceptable way to receive health services
   - I am overall satisfied with the experience I have had in telemedicine
2. Indicate which of these benefits you believe you have benefited most thanks to the telemedicine experience with the rheumatology department (you can choose more than one):
   - Better medical treatment experience
   - Saving of travel time
   - Saving of waiting time
   - Immediate and direct communication through telephone contact with healthcare professionals
   - Convenience for the home delivery of drugs
   - Greater emotional and psychological support received from medical staff
   - Other ________
3. Indicates on a scale from 1 (disagree) to 5 (completely agree) which of these channels you would like to use in the future to receive health services from the rheumatology department:
   - Video call with your reference doctor
   - Phone call from medical staff
   - Phone call from nursing staff
   - Home delivery of drugs
   - Interactions by e-mails or messages on the telephone with medical staff
   - Other ___________
4. If the rheumatology department reproposed you a telemedicine experience, how would you behave?
   - I would definitely accept
   - Maybe I would accept
   - I don't think I would accept
   - I definitely wouldn't accept
5. **(for those who answered question 24 with "I would definitely accept" or "Maybe I would accept")** If the rheumatology department riproposed you a telemedicine experience, please indicate what you would prefer:
   - Keep the current frequency of access to the department, but with the possibility of also using telemedicine services
   - Reduce the current frequency of access to the ward, replacing some accesses with telemedicine services
   - Use only telemedicine services, without going to hospital
   - Other __________

**Questionnaire reserved for non-users (identified by question 19)**

To answer the next questions, imagine being able to have a telemedicine experience with the rheumatology department. The telemedicine services that could be offered to you, in partial replacement of the services offered in person, consist of contacts with the rheumatology facility through technological support.

1. On a scale of 1 (disagree) to 5 (completely agree), indicate what you think of the following statements:
   - It was easy to use the system to fill the questionnaires at home
   - It was easy to learn how to use the system to fill the questionnaires at home
   - It would be easy for me to fill the questionnaires at home, through the use of technological devices (eg. App, telephone, computer, tablet) in which I provide information on how I feel
   - Telemedicine can improve access to health services
   - Telemedicine would allow me to receive specialized care that I otherwise would not have been able to access
   - Telemedicine would save me the travel time to go to the hospital
   - Telemedicine would meet my health needs
   - I believe that communications with staff through the telemedicine system would be equivalent to communications in face-to-face meetings
   - I would enjoy communicating with medical staff using telemedicine tools
   - I believe that telemedicine tools would improve my ability to talk to doctors (eg. Asking questions, telling concerns, etc.)
   - Telemedicine is a way that I would reckon acceptable to receive health services
2. Indicate which of these benefits you believe you woul benefit most by joining a telemedicine experience (you can choose more than one):
   - Better medical treatment experience
   - Saving of travel time
   - Saving of waiting time
   - Immediate and direct communication through telephone contact with healthcare professionals
   - Convenience for the home delivery of drugs
   - Greater emotional and psychological support received from medical staff
   - Other ________
3. Indicates on a scale from 1 (disagree) to 5 (completely agree) which of these channels you would like to use in the future to receive health services from the rheumatology department:
   - Video call with your reference doctor
   - Phone call from medical staff
   - Phone call from nursing staff
   - Home delivery of drugs
   - Interactions by e-mails or messages on the telephone with medical staff
   - Other ___________
4. If the rheumatology department proposed you a telemedicine experience, how would you behave?
   - I would definitely accept
   - Maybe I would accept
   - I don't think I would accept
   - I definitely wouldn't accept
5. **(for those who answered question 23 with "I would definitely accept" or "Maybe I would accept")** If the rheumatology department proposed you a telemedicine experience, please indicate what you would prefer:
   - Keep the current frequency of access to the department, but with the possibility of also using telemedicine services
   - Reduce the current frequency of access to the ward, replacing some accesses with telemedicine services
   - Use only telemedicine services, without going to hospital
   - Other __________

**Appendix 3. Checklist for Reporting Of Survey Studies (CROSS)**

| **Section/topic** | **Item** | **Item description** | **Reported on page #** |
| --- | --- | --- | --- |
| **Title and abstract** | | |  |
| Title and abstract | 1a | State the word “survey” along with a commonly used term in title or abstract to introduce the study’s design. | 1 |
|  | 1b | Provide an informative summary in the abstract, covering background, objectives, methods, findings/results, interpretation/discussion, and conclusions. | 1-2 |
| **Introduction** | | |  |
| Background | 2 | Provide a background about the rationale of study, what has been previously done, and why this survey is needed. | 3-4 |
| Purpose/aim | 3 | Identify specific purposes, aims, goals, or objectives of the study. | 3-4 |
| **Methods** | | |  |
| Study design | 4 | Specify the study design in the methods section with a commonly used term (e.g., cross-sectional or longitudinal). | 4 |
|  | 5a | Describe the questionnaire (e.g., number of sections, number of questions, number and names of instruments used). | 5-6-7 |
| Data collection methods | 5b | Describe all questionnaire instruments that were used in the survey to measure particular concepts. Report target population, reported validity and reliability information, scoring/classification procedure, and reference links (if any). | 5-6-7 |
|  | 5c | Provide information on pretesting of the questionnaire, if performed (in the article or in an online supplement). Report the method of pretesting, number of times questionnaire was pre-tested, number and demographics of participants used for pretesting, and the level of similarity of demographics between pre-testing participants and sample population. |  |
|  | 5d | Questionnaire if possible, should be fully provided (in the article, or as appendices or as an online supplement). | Appendix 2 |
| Sample characteristics | 6a | Describe the study population (i.e., background, locations, eligibility criteria for participant inclusion in survey, exclusion criteria). | 7 |
|  | 6b | Describe the sampling techniques used (e.g., single stage or multistage sampling, simple random sampling, stratified sampling, cluster sampling, convenience sampling). Specify the locations of sample participants whenever clustered sampling was applied. | 7 |
|  | 6c | Provide information on sample size, along with details of sample size calculation. | 9 |
|  | 6d | Describe how representative the sample is of the study population (or target population if possible), particularly for population-based surveys. | 9 |
| Survey  administration | 7a | Provide information on modes of questionnaire administration, including the type and number of contacts, the location where the survey was conducted (e.g., outpatient room or by use of online tools, such as SurveyMonkey). | 7 |
|  | 7b | Provide information of survey’s time frame, such as periods of recruitment, exposure, and follow-up days. | 7 |
|  | 7c | Provide information on the entry process:  –>For non-web-based surveys, provide approaches to minimize human error in data entry.  –>For web-based surveys, provide approaches to prevent “multiple participation” of participants. | 7 |
| Study preparation | 8 | Describe any preparation process before conducting the survey (e.g., interviewers’ training process, advertising the survey). |  |
| Ethical considerations | 9a | Provide information on ethical approval for the survey if obtained, including informed consent, institutional review board [IRB] approval, Helsinki declaration, and good clinical practice [GCP] declaration (as appropriate). | 7 |
|  | 9b | Provide information about survey anonymity and confidentiality and describe what mechanisms were used to protect unauthorized access. | 7 |
| Statistical  analysis | 10a | Describe statistical methods and analytical approach. Report the statistical software that was used for data analysis. | 7-8 |
|  | 10b | Report any modification of variables used in the analysis, along with reference (if available). | 8 |
|  | 10c | Report details about how missing data was handled. Include rate of missing items, missing data mechanism (i.e., missing completely at random [MCAR], missing at random [MAR] or missing not at random [MNAR]) and methods used to deal with missing data (e.g., multiple imputation). | 9 |
|  | 10d | State how non-response error was addressed. | 9 |
|  | 10e | For longitudinal surveys, state how loss to follow-up was addressed. |  |
|  | 10f | Indicate whether any methods such as weighting of items or propensity scores have been used to adjust for non-representativeness of the sample. |  |
|  | 10g | Describe any sensitivity analysis conducted. |  |
| **Results** | | |  |
| Respondent characteristics | 11a | Report numbers of individuals at each stage of the study. Consider using a flow diagram, if possible. | 9 |
|  | 11b | Provide reasons for non-participation at each stage, if possible. | 9 |
|  | 11c | Report response rate, present the definition of response rate or the formula used to calculate response rate. | 9 |
|  | 11d | Provide information to define how unique visitors are determined. Report number of unique visitors along with relevant proportions (e.g., view proportion, participation proportion, completion proportion). |  |
| Descriptive  results | 12 | Provide characteristics of study participants, as well as information on potential confounders and assessed outcomes. | 9-10 |
| Main findings | 13a | Give unadjusted estimates and, if applicable, confounder-adjusted estimates along with 95% confidence intervals and p-values. | 10-11-12-13-14-15 |
|  | 13b | For multivariable analysis, provide information on the model building process, model fit statistics, and model assumptions (as appropriate). | 14 |
|  | 13c | Provide details about any sensitivity analysis performed. If there are considerable amount of missing data, report sensitivity analyses comparing the results of complete cases with that of the imputed dataset (if possible). |  |
| **Discussion** | | |  |
| Limitations | 14 | Discuss the limitations of the study, considering sources of potential biases and imprecisions, such as non-representativeness of sample, study design, important uncontrolled confounders. | 19 |
| Interpretations | 15 | Give a cautious overall interpretation of results, based on potential biases and imprecisions and suggest areas for future research. | 16-17-18-19 |
| Generalizability | 16 | Discuss the external validity of the results. | 19 |
| **Other sections** | | |  |
| Role of funding source | 17 | State whether any funding organization has had any roles in the survey’s design, implementation, and analysis. | 22 |
| Conflict of interest | 18 | Declare any potential conflict of interest. | 22 |
| Acknowledgements | 19 | Provide names of organizations/persons that are acknowledged along with their contribution to the research. | 22-23 |
